# Marine Omega-3 Phospholipids: Metabolism and Biological Activities

**DOI:** 10.3390/ijms131115401

**Published:** 2012-11-21

**Authors:** Lena Burri, Nils Hoem, Sebastiano Banni, Kjetil Berge

**Affiliations:** 1 Aker BioMarine ASA, Fjordalléen 16, NO-0115 Oslo, Norway; E-Mails: lena.burri@akerbiomarine.com (L.B.); nils.hoem@akerbiomarine.com (N.H.); 2 Department of Biomedical Sciences, University of Cagliari, Cagliari, Italy; E-Mail: banni@unica.it

**Keywords:** phospholipid, krill oil, phosphatidylcholine, fish oil, triglyceride

## Abstract

The biological activities of omega-3 fatty acids (n-3 FAs) have been under extensive study for several decades. However, not much attention has been paid to differences of dietary forms, such as triglycerides (TGs) versus ethyl esters or phospholipids (PLs). New innovative marine raw materials, like krill and fish by-products, present n-3 FAs mainly in the PL form. With their increasing availability, new evidence has emerged on n-3 PL biological activities and differences to n-3 TGs. In this review, we describe the recently discovered nutritional properties of n-3 PLs on different parameters of metabolic syndrome and highlight their different metabolic bioavailability in comparison to other dietary forms of n-3 FAs.

## 1. Introduction

Marine omega-3 phospholipids (n-3 PLs) are defined as PLs containing n-3 long-chain polyunsaturated fatty acids (PUFAs) derived from marine organisms. This makes them different from PLs derived from vegetable sources, since they do not contain long-chain n-3 PUFAs.

A substantial number of studies on n-3 FAs have assessed their health benefits [1,2]. Research on beneficial effects of n-3 PUFAs against atherosclerosis and thrombosis was initiated by studies done by Dyerberg and Bang on the Greenland Eskimos in the early 1970s [3,4]. Later, n-3 PUFAs have been shown to be crucial at cellular levels for maintaining membrane homeostasis, as ligands for transcription factors thus influencing gene expression, and for an optimal balance with n-6 fatty acids to secure a regulated inflammatory response, a healthy body composition homeostasis and an equilibrated neurological and psychiatric balance [5,6].

Most of the clinical studies have been carried out using n-3 FAs bound to TGs or ethyl esters. However, more recently, marine products enriched in n-3 PUFAs bound to PLs have received increasing attention for their emerging health benefits, as discussed in this review.

## 2. PL Classes

**Figure 1:**
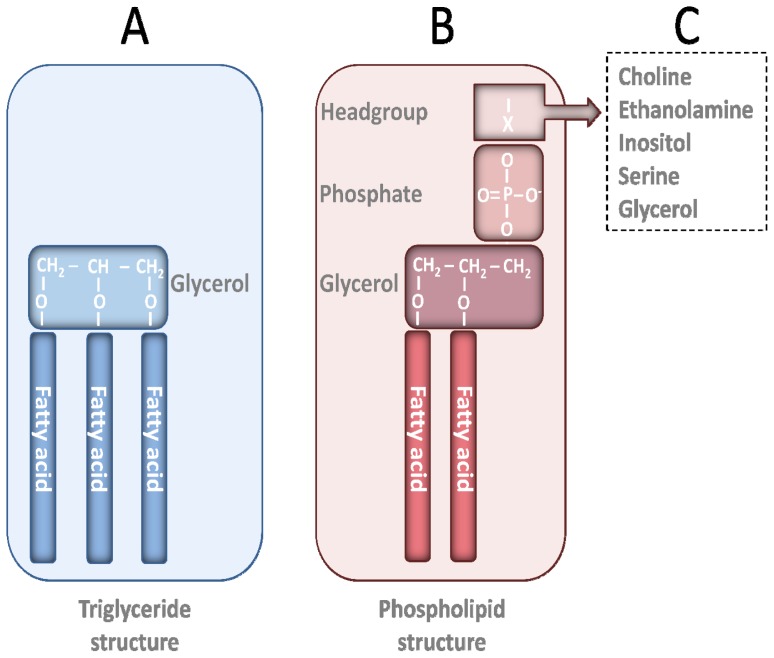
Triglyceride and phospholipid structures.

In nature, n-3 FAs are mainly esterified either in PLs or TGs or, due to a partial hydrolysis, are present in the free form. Whereas TGs consist of three FAs esterified to a glycerol backbone, PLs have normally two FAs esterified to a glycerol backbone, and a phosphorous group that is further linked to a headgroup (Figure 1). TGs are highly hydrophobic, whereas PLs are hydrophilic due to the polar headgroup. Hence, the physical-chemical properties of these two groups of lipid structures are different and only PLs are able to form micelles and liposomes.

There are three main classes of PLs: glyceroPLs (FAs ester-bound to backbone), ether glycerolipids and sphingoPLs. GlyceroPLs are the most common subgroup and can be further divided into different subgroups, which are defined by the head-group located in the PL (Figure 1). The headgroup can consist of choline, ethanolamine, glycerol, inositol or serine. For example, phosphatidylcholine (PC) contains a choline head-group, whereas phosphatidylethanolamine (PE) contains an ethanolamine head-group. The distribution of the different PLs in marine resources was recently reviewed by Lu and colleagues [7]. According to this review, the most predominant PLs in marine sources such as salmon, tuna, rainbow trout and mackerel, is PC, whereas PE is shown to be the second most abundant. Phosphatidylinositol (PI), phosphatidylserine (PS), lysophosphatidylcholine (lyso-PC), and sphingomyelin are found in smaller amounts.

## 3. Sources of Marine PLs

### 3.1. Fish Roe

One by-product of the fish industry that is particularly interesting in respect to marine PLs is fish roe. The word ‘roe’ stands for the eggs and the ovaries full of eggs of seafood. Fish roe is used for human consumption and is a rich source of n-3 PUFAs in PL form [8–10]. Fish roe from herring, salmon, pollock, and flying fish contain between 38%–75% of their lipids in the form of PLs with PC being the predominant lipid class [11]. In salmon, that had the highest total lipid content of the four fish examined, 56% of the lipids were in TG form, whereas the other roes had values below 20%. More than 30% of the total FAs were eicosapentaenoic acid (EPA, 20:5n*-*3) or docosahexaenoic acid (DHA, 22:6n-3). A high presence of these two FAs in fish roes has also been demonstrated in other studies [8,12,13].

### 3.2. Krill Oil

One important source of marine PLs is krill oil (KO). KO has become increasingly popular as a food supplement during the last decade, and the research on KO in animal and human studies is growing. KO is extracted from the Antarctic crustacean krill (*Euphausia superba*), a shrimp-like zooplankton. It is known for a high amount of PL-bound n-3 PUFAs, in particular EPA and DHA. The majority of these two fatty acids are contained in the PC form [14,15]. The amount of PLs in oil extracted from krill is typically around 40%. The FA composition of the PLs in KO was recently characterized in two studies [16,17]. Both studies revealed a complex composition of PL species in KO. In the study performed by Winther *et al*., 69 different choline-containing PL molecules were identified. Of these, 58% contained n-3 PUFA in one position, whereas n-3 PUFAs in both positions were found in 10% of the PC molecules. The most prevalent n-3 PUFAs were EPA and DHA, but also stearidonic acid and docosapentaenoic acid (DPA) were present in smaller amounts.

### 3.3. Fish

The main dietary source of EPA and DHA is fish, in particular cold-water oily fish like salmon, sardine, anchovy, herring, or mackerel. Fish contains between 1%–1.5% PLs and 10%–15% TGs [18]. Depending on the kind of fish, up to one third of the EPA and DHA content might exist in the form of PLs [19]. One study has shown that in Atlantic salmon, EPA and DHA are bound to PLs and TGs in a 40:60 ratio [20]. Hence, fish represents a potential source of marine PLs, but the production of marine PLs from fish has so far been limited. Moreover, the amount of by-products from fish is significant and thus further represents a valuable source of marine PLs.

However, the FA content and also the overall amount of fat in farmed fish can be quite different in comparison to wild fish. Because vegetable oils and oilseeds are used in aquaculture feed, the (EPA + DHA) to (arachidonic acid + linolenic acid) ratio can be as low as 1.5 for farmed *versus* 14.5 for wild salmon [21]. In comparison, the corresponding ratio in KO is 12.0 [22].

## 4. Different Tissue Distribution of FAs from PL and TG Ester Forms

The FA chain length and unsaturation affect the intestinal absorption efficiency of lipids, whereas the chemical structure of the lipids (TGs *versus* PLs) determines their digestion products before absorption [23–25]. Digestion of TGs was suggested to start in the stomach, where they are hydrolyzed by lingual and gastric lipases. However, only insignificant amounts of PLs are substrates for these enzymes and the major portion of PLs is hydrolyzed in the small intestine by pancreatic phospholipase A_2_ with the help of other lipases. In contrast to the hydrolysis products of TGs, the corresponding products from PLs are not dependent on bile salts to form micelles. After uptake into the enterocytes, the PLs are mainly incorporated into the chylomicron surface layer, while the TGs are solubilized in the interior of chylomicrons [24]. There are, however, studies with human intestinal CaCo-2 cells [26], rat [27] and swine [28] suggesting that a substantial part of the dietary PL fraction is integrated into high-density lipoprotein (HDL) particles already in the intestine that later join the plasma HDL pool. In addition, others have suggested an important role for the intestine in the synthesis of HDL [29–31].

In the bloodstream, chylomicrons are catabolized by the endothelial-bound enzyme lipoprotein lipase (LPL), which hydrolyzes TGs, releasing FAs, which can subsequently be taken up by tissue [32]. As a result of delipidation of chylomicrons, chylomicron remnant particles are formed, which are finally taken up and metabolized by the liver. During circulation in the blood stream, lipid components of chylomicrons can also be transferred to HDL, which is a process catalyzed by cholesteryl ester transfer protein (CETP) that exchanges cholesteryl esters from HDL to the chylomicron remnant particles and TGs to HDL [33].

FA absorption in the small intestine has been found equivalent for both the PL- and TG-derived compounds [34–38]. It has, however, been proposed that the different PL and TG ester forms could determine in which blood pool (free FAs, outer *versus* inner layer of chylomicrons, different lipoprotein particles) the FAs end up [39]. This could then influence the distribution of FAs into different tissues. There is some evidence that PLs might be a more efficient delivery form of PUFAs to the brain than TGs [40,41]. For example, it has been found by radioactive labeling of DHA in PC and TG esters that DHA from PC is more efficiently taken up into different tissues than DHA from TG in ten week old rats [42]. The incorporation of DHA was more than twice as high from PLs as from TGs in brain, liver, and kidney, and higher DHA incorporation was also seen in adrenal gland, brown fat, thymus and ulveal tract/retina. More specifically, in the brain the DHA uptake was significantly increased in 11 out of 14 brain regions after PL administration, compared to supplementation in TG form. The increased tissue accumulation of radioactivity in the PC form became apparent only in ten weeks old animals and was absent in two week old animals. Another observation of the study was a strong decrease of ^14^C-DHA coming from either ester form in the brain in older animals compared to younger animals.

A further study has equally found that radiolabeled arachidonic acid esterified in PLs accumulates more efficiently in baboon neonate tissues (brain, liver and lung) than do the TG structures [43]. The incorporation of arachidonic acid into the brain tissue was 2.1 fold higher when baboons were supplemented with PLs, as compared to TG administration. Moreover, a study in obese Zucker rats compared n-3 FAs either given in the form of fish oil (FO; n-3 FAs in TGs) or KO (majority of n-3 FAs in PLs) [44]. In this study, KO led to a significantly higher incorporation of EPA and DHA into tissue PLs. In heart, there was a 96% and 42% higher incorporation of EPA and DHA, respectively, into the PLs after KO compared to FO supplementation. Similar observations were identified in the liver. EPA and DHA levels were 47% and 13% higher after KO administration, which is in agreement with the studies in rats and baboons [42,43]. There was also a significantly higher presence of DHA in the brain after KO supplementation to the diet as compared to FO supplementation [45]. This finding was supported by a recent study with KO demonstrating increased DHA amount in the brain and beneficial effect on memory function in rats [46]. Moreover, it has been demonstrated that DHA in the form of lyso-PC, and more specifically, when DHA is located in the *sn*-1 position, is efficiently taken up in the developing rat brain [47]. Also other studies performed by the same research group have documented that lyso-PC can be an effective delivery form of DHA to the rat brain [48]. In addition, Cansell and colleagues have investigated the metabolism of EPA and DHA delivered either in PL (liposomes) or TG form [49–52]. They found an increased n-3 PUFA bioavailability from PLs in the lymph, in comparison to n-3 PUFAs from TGs. Numerous studies in humans, such as the Framingham study, linked DHA levels in blood plasma to brain-related disorders like Alzheimer’s disease, and several different protective roles of DHA in the brain have been suggested [53,54]. Finally, in humans it has been demonstrated that PLs are a more efficient delivery form of DHA to platelets and erythrocytes than TGs [55].

The mechanism behind an increased tissue uptake of n-3 PUFAs bound to PLs is currently not known, and more detailed mechanistical studies need to be done in order to fully explain the differences in tissue accumulations between the two ester forms.

## 5. Health Effects of Non-Marine and Marine PLs

Due to the dual hydrophilic and hydrophobic properties of PLs, they are mainly known for their role as building blocks for cell membranes in almost all known living beings. In addition to their role in cellular structure and function, they play an important part in the formation of lipoproteins, which transport lipids to tissues via the blood stream. Additionally, certain PL metabolites serve as important molecules within several signaling systems.

During the latest years, more and more focus has been laid on the beneficial health effects of PLs in both animals and humans. Marine n-3 PLs however, that come in a particular blend of PL molecules and n-3 PUFAs bound to it, have been studied to a lesser extent. However, during the last decade several papers have described the effects of two important sources of marine PLs: KO and fish roe, which is summarized in Section 5.4 (n-3 PLs) below. The studies with marine PLs in humans and animals are listed in Tables 1 and 2.

### 5.1. PLs

PCs by themselves, without the added benefit of n-3 PUFAs, have been described to alleviate senescence [80] and to be beneficial for cognitive functions [81], inflammatory diseases [82], as well as plasma and liver lipid metabolism [83,84]. Other studies have shown that PLs might increase sport performance [85,86]. The beneficial effects of PLs on blood and hepatic lipids have been studied in a number of animal experiments [83,87–94], and both cholesterol and TG levels are affected upon treatment. In particular, egg and soybean PLs have been shown to increase levels of HDL in humans [84] and to lower hepatic TG levels by 45% in orotic acid-induced fatty liver in rats [83]. In addition, trials in humans have demonstrated that PLs can protect the liver against alcohol-related damages [95–97]. PC was further described by Lieber and colleagues to defend against liver fibrosis and cirrhosis induced by alcohol fed to baboons over a period of 6.5 years [12]. Interestingly, patients with fatty liver showed a PL profile modification with a decrease of PC and PE [98].

Three underlying mechanisms seem to be important for the beneficial effects on cholesterol and/or TG after PL administration: (a) increased sterol excretion, (b) stimulated lipid secretion from bile, and (c) increased β-oxidation and decreased synthesis of lipids in the liver [99]. The majority of the studies performed with PLs did not include n-3 PUFAs-containing PLs, indicating that PLs in general have beneficial effects. However, other studies have demonstrated that PLs containing n-3 PUFAs have more potent effects on liver and blood plasma lipid levels, compared to PLs without n-3 PUFAs [94,100].

### 5.2. Choline

Choline, the head-group of PC and sphingomyelin, is an essential human nutrient. Rich sources of choline are beef liver, egg, fish, chicken and milk [101]. KO, which contains approximately 40% of PC, is also a good source of choline. In addition to being an important part of PC and to being a major source of methyl groups in the *S*-adenosylmethionine pathway, choline is also a substrate in the synthesis of acetylcholine. This neurotransmitter is involved in neuron networks associated with memory [102]. Aging may depress the availability of neurotransmitters like acetylcholine. Hence, it has been hypothesized by some researchers that supplementation of choline-containing compounds like PC might stimulate the production of acetylcholine and have a beneficial impact on the central nervous system [103].

Choline supplementation is especially important for vegetarians, vegans and people who drink excess amounts of alcohol, since they have an elevated risk of choline deficiency. In addition to an increased risk of developing liver dysfunction [12,104,105], choline deficiency might also hinder normal development of memory function [106]. Supplementation with choline has been attributed to impact on brain function, when given to rats either before birth or during the second week after birth [13,107–111]. The long-lasting effect of choline administration on rodent spatial memory function seems to involve changes in the hippocampus [112–114]. There is also some evidence that a high intake of choline might reduce the risk of breast cancer and colorectal cancer [115–117].

### 5.3. n-3 PUFAs

The main source of n-3 PUFAs are algae that can synthetize them *de novo*. They are the basis for the enrichment of n-3 PUFAs in the lipids of other marine organisms over the aquatic food chain. The n-3 FAs distinguish themselves from the n-6 FAs by the position of the first double bond from the methyl end of the chain. N-6 metabolites cannot be converted into n-3 FAs and humans can only poorly synthesize n-3 PUFAs from their precursor α-linolenic acid (ALA; 18:3n*-*3), which is present in plants like rapeseed, soybean, walnut, flaxseed, perilla, chia, and hemp. The dietary intake of EPA and DHA is essential as they are extensively associated with optimal human health and protection against disease.

EPA and DHA have the ability to influence cell membrane fluidity, permeability or membrane protein-mediated responses. By being precursors to lipid mediators (eicosanoids/docosanoids) or as ligands for transcription factors, these n-3 PUFAs affect cell and tissue physiology and response to external signals [118]. By these means, EPA and DHA support cardiovascular health as well as cognitive, visual, immune, and reproductive system functions. There are also indications that they confer health benefits regarding e.g. tumorigenesis, hypertriglyceridemia, atherosclerosis, mental illness, dementia, bone health, and attention-deficit hyperactivity disorder (ADHD) [119–130]. In spite of an overwhelming amount of evidence on the beneficial effects of n-3 PUFAs on human health, some controversy remains [131–135]. The reason might be that published meta-analysis were based on different study inclusion criteria, e.g., number of subjects, length of treatment, n-3 dose given, disease state of participants, co-medications, compliance of study product intake, dietary background (intake of fish/other n-3 products), placebo/not placebo controlled, *etc.* This led to a significant heterogeneity in conclusions. Moreover, there is a variation in statistical strictness (*p*-values) used in the different analysis. Nevertheless, the totality of the scientific research supports a cardiovascular benefit of n-3 PUFAs in healthy individuals, as well as in several groups with pre-existing cardiovascular diseases.

### 5.4. n-3 PLs

#### 5.4.1. KO

Intake of n-3 PUFAs in the chemical form of PLs from KO has been associated with several health promoting aspects (Tables 1 and 2). In some studies, the effects were stronger than the ones observed with FO, which may be attributed to an increased efficacy of EPA and DHA in PL form compared to the TG form and to their different target tissue accumulations [22,63,64]. Two studies in obese animals have demonstrated that KO has beneficial effects on fatty liver and plasma cholesterol levels [44,70]. In the diet-induced obesity model it was also demonstrated that KO improves insulin sensitivity [70]. Further, KO has been shown to have effects on markers of inflammation and arthritis symptoms in preclinical and clinical studies [58,75]. In addition, beneficial changes in hepatic and cardiac steatosis, plasma hypercholesterolemia, glucose homeostasis, adiponectin levels, and premenstrual syndrome have been associated with the intake of KO in several studies [22,56,60,70]. Moreover, increased endocannabinoid levels, as metabolic parameters of metabolic syndrome and obesity [136], have been found to be ameliorated in obese rats, mice and humans after KO supplementation [44,45,57,69]. Other recent studies have demonstrated that KO improves memory function, protects against heart failure and attenuates inflammation in ulcerative colitis in rats [46,65,74].

#### 5.4.2. Fish and Fish Roe

A recent study comparing n-3 TGs and n-3 PLs (isolated from fish) revealed that the PL form was better in improving the metabolic profile in obese mice than the TG form [67]. EPA and/or DHA were shown to be more efficiently taken up in tissues like liver, white adipose tissue and muscle, when given in the PL form. The beneficial effect of PLs on plasma insulin and adipose hypertrophy was partly explained by their effect on the endocannabinoid metabolome in white adipose tissue. A number of reports have looked at the health benefits of various fish roes in animals and humans. In mice, the intake of extracted herring roe lipids led to an increase in plasma adiponectin levels and a decrease in plasma TG, PL, total cholesterol, and glucose amounts [8]. The same authors have also shown in a maze-behavior experiment that the extracted lipids improve learning capacity in mice [9]. Three other studies using n-3 rich PLs from herring roe have further demonstrated beneficial effects in different animal models [8,9,66,71]. In obese Otsuka Long-Evans Tokushima fatty rats, an animal model used for human obesity, PC extracted from salmon roe led to a reduction in liver weight and hepatic lipid levels after four weeks of administration [94]. The changes were attributed to the suppression of FA synthesis and increase of FA β-oxidation.

In a small human trial of six patients, PC obtained from salmon roe were tested for their potential to alleviate chronic liver disease during six month of administration [10]. Results showed significantly decreased serum globulin and increased HDL and apolipoprotein A–I and E levels. However, no other blood parameters important for liver function were affected and a larger and longer study design might help to fully unveil the potential of n-3 PLs in improving hepatic damage and inflammation. Another human study over six weeks showed that marine PLs from salmon roe might help in the prevention of tumor-associated weight loss [137]. Daily, the patients received 1.5 g marine PLs for six weeks and a stabilization of body weight was achieved in addition to improved appetite and quality of life.

## 6. Future Perspectives

Commercial PLs produced from vegetable sources are usually processed from soybean oil (also called soy lecithin), and are widely used as functional ingredients in pharmaceutical industries, cosmetics, and food. PLs are also processed from non-marine animal sources like egg, milk and brain, or marine sources like krill, fish by-products, and roe.

There are two main areas of pharmaceutical applications of marine PLs: (1) nutritional use, and (2) drug delivery. Within drug delivery, PLs are of importance, since they are able to form liposomes after mixture into an aqueous media. Liposomes have been investigated as drug carriers for decades and several different types have been developed.

The second important area for the use of PLs is within cosmetics, where their applications include emollients and specific ingredients for skin care, hair care, make-up and decorative products. The emulsifying properties of PLs are used in skin moisturizing products, where they play two important roles. In addition to having potential beneficial properties for the epidermis, they are also used as vehicles or carriers in dermatologic delivery systems.

PLs play an important role in food manufacturing and are currently used in a wide range of food products such as baked goods, instant drinks, dairy products, chocolate and margarine. The most widely used application of PLs in foods is as an emulsifier, where it is utilized in both water-in-oil emulsions, as well as in oil-in-water emulsions. However, modern diets are depleted in PLs due to increased use of refined oils and purified raw materials, which had led to a lower intake of PLs. Hence, the supplementation of marine PLs may serve three important functions within the functional food segment: (a) emulsifying properties, (b) supplementation of n-3 FAs and (c) beneficial nutritional effects of the PLs themselves.

Traditional PLs used within nutrition or for pharmaceutical and cosmetic applications do not normally contain n-3 PUFAs, or at best only very small amounts. The increased accessibility of marine PLs during recent years opens up new possibilities for the use of PLs not only as a superior nutritional source of n-3 PUFAs, but also for use in the pharmaceutical, cosmetic and functional food industries.

## Figures and Tables

**Table 1:** Overview over clinical studies with marine n-3 PLs.

Area of study	Population characteristics	Treatment	Main findings	References
Cardiovascular	Dyslipidemia	KO	Improved blood lipids	[56]
Obesity	Normal to obese	KO	Changed endocannabinoid levels	[57]
Inflammation	Arthritis	KO	Reduced arthritic symptoms	[58]
	Athletes	KO	Reduced oxidative damage	[59]
PMS	Women	KO	Reduced dysmenorrhea	[60]
Brain	Memory complains	n-3 PS 1	Improved word recall	[61]
Eye	ADHD children	n-3 PLs 2	Improved attention	[62]
Liver	Chronic liver disease	Roe 3	Improved lipid parameters	[10]
Bioavailability	Healthy	KO	Increased n-3 FA blood levels	[22,63,64]

FA, fatty acid; KO, krill oil; PLs, phospholipids; PMS, premenstrual syndrome; PS, phosphatidylserine.

1 The n-3 PS was synthesized from krill.

2 The n-3 PLs were isolated from KO.

3 Salmon roe.

**Table 2:** Overview over pre-clinical studies with marine n-3 PLs.

Area of study	Animal model	Treatment	Main findings	References
Cardiovascular	Heart failure (r)	KO	Attenuated heart remodeling	[65]
	Healthy (m)	Roe 1	Improved blood lipids and adiponectin	[8,66]

Obesity	High fat diet (m)	n-3 PLs 2	Improved metabolic profile	[67]
	High-fat diet (r)	KO	Decreased body weight	[68]
	High-fat diet (m)	KO	Reduced endocannabinoid biosynthesis	[69]
	High-fat diet (m)	KO	Decreased hepatic steatosis	[70]
	Genetic obesity (r)	KO	Decreased hepatic and heart lipids	[44]
	High-fat diet (m)	Roe 3	Reduced abdominal fat	[71]

Inflammation	TNFα overexpression (m)	Roe 3, KO	Increased hepatic β-oxidation	[72–74]
	Ulcerative colitis (r)	KO	Reduced oxidative stress	[75]
	Arthritis (m)	KO	Reduced arthritis scores	

Brain	Healthy (r)	n-3 PLs 4	Improved memory function	[46]
	Genetic obesity (r)	KO	Increased DHA level in brain	[45]
	Healthy (r)	n-3 PS 5	Improved learning and memory	[76]
	Healthy (m)	Roe 1	Improved learning capacity	[9]

Bone	Growing females (r)	KO	Did not improve bone mass/architecture	[77]

Other	Healthy (r)	KO	Decreased hepatic lipogenesis	[78]
	Healthy (m)	KO	Beneficial hepatic gene regulation	[79]

(m), mice; (r), rats; KO, krill oil; PLs, phospholipids; PS, phosphatidylserine.

1 Salted herring roe.

2 Extracted from herring meal.

3 Herring roe.

4 Isolated from KO.

5 Synthesized from KO.
